# 500. Finding AP Gap: An Assessment of Antibiotic Use in Acute Pancreatitis

**DOI:** 10.1093/ofid/ofae631.152

**Published:** 2025-01-29

**Authors:** Emily Brassell, Andrea H Son, Michelle T Hecker

**Affiliations:** MetroHealth Medical Center, Columbus, OH; The MetroHealth System, Cleveland, Ohio; The MetroHealth System, Cleveland, Ohio

## Abstract

**Background:**

American Gastroenterology Association (AGA) guidelines recommend against the use of prophylactic antibiotics for acute pancreatitis. Antibiotic therapy is recommended for culture-proven infection in pancreatic necrosis or when infection is strongly suspected (e.g. gas in collection, bacteremia, sepsis, or clinical deterioration). Carbapenems, metronidazole, third or fourth generation cephalosporins, and fluoroquinolones are preferred agents. Data to support use of piperacillin/tazobactam also exists. There is limited data evaluating adherence to these guidelines.

**Table 1:**
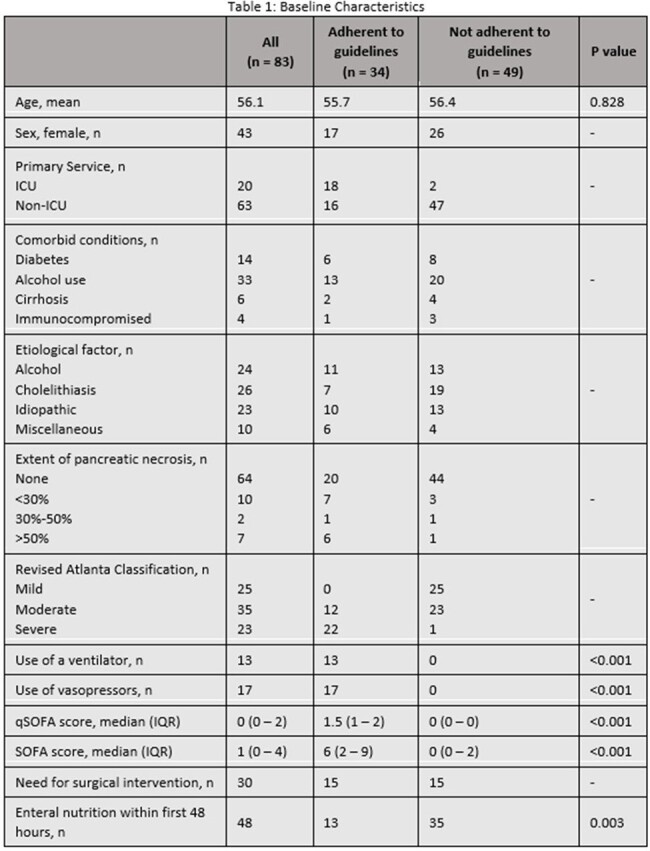
Baseline Characteristics

**Methods:**

This was a retrospective cohort study of adult patients hospitalized in The MetroHealth System from January 1, 2021 through December 31, 2022 who received antibiotics for a diagnosis of acute pancreatitis. Exclusion criteria included multiple indications for antibiotics or antibiotics given in the emergency department (ED) only. Only the first hospitalization per patient was included. The primary outcome was the percentage of encounters for which antibiotic use was adherent to AGA guidelines. Secondary outcomes included descriptions of antibiotic choice/duration and microbiologic results.

**Table 2:**
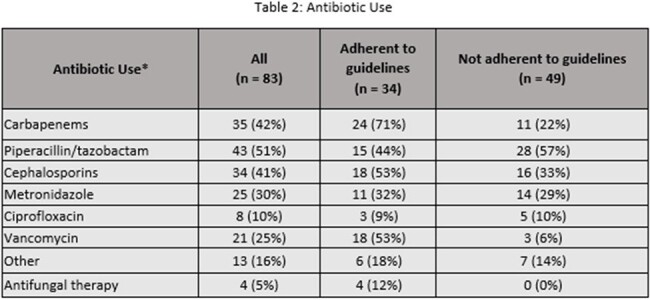
Antibiotic Use

**Results:**

Of 538 inpatient encounters with a diagnosis of acute pancreatitis, antibiotics for any indication were prescribed in 322 (60%). For the 83 unique patient encounters meeting inclusion criteria, antibiotic use was not adherent to AGA guidelines in 49 (59%). Table 1 compares characteristics of patients in whom antibiotic therapy was adherent vs non-adherent. Carbapenems, piperacillin/tazobactam, cephalosporins, and vancomycin were prescribed for pancreatitis during 42%, 51%, 41%, and 25% of encounters respectively (table 2). The average duration of antibiotics for all study patients was 8.5 days. Microbiologic testing was performed in 64 (77%) patients with no MRSA isolated in any cultures (Table 3).

**Table 3:**
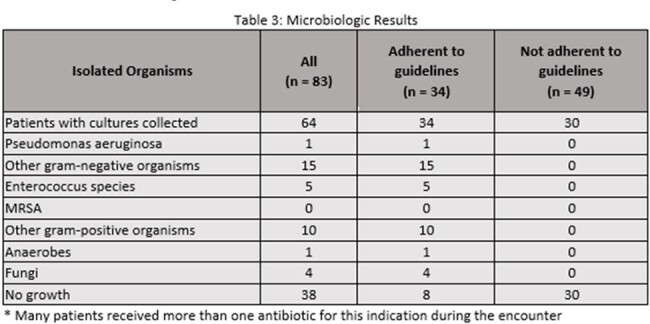
Microbiologic Results

**Conclusion:**

Antibiotic use in more than half of patients was not adherent to AGA guidelines. Carbapenems, piperacillin/tazobactam, and cephalosporins were commonly used. Vancomycin was often used, although no MRSA was isolated. Significant opportunities for antimicrobial stewardship for acute pancreatitis exist.

**Disclosures:**

**All Authors**: No reported disclosures

